# The sense of guilt of the mothers of children with special needs

**DOI:** 10.1192/j.eurpsy.2022.607

**Published:** 2022-09-01

**Authors:** E. Sedova, E. Tokmakova, T. Goryacheva, Z. Gardanova

**Affiliations:** 1 Pirogov Russian National Research Medical University, Faculty Of The Clinical Psychology And Social Work, Moscow, Russian Federation; 2 Pirogov Russian National Research Medical University, Faculty Of The Clinical Psychology And Social Work, Москва, Russian Federation

**Keywords:** sense of guilt, children with special needs

## Abstract

**Introduction:**

The fact of emergence of a child with special needs in a family is followed by intensive emotional reaction of the parents. One of the pronounced feelings in those circumstances is the sense of guilt.

**Objectives:**

The research aim is studying the emotional experience of mothers of children with special needs connected with the sense of guilt and the characteristics of the parent-child relationship in such families.

**Methods:**

The research sample includes 25 mothers of children with special needs in the age from 3 to 10 years old with the diagnosis ASD, cerebral palsy and epilepsy as well as 29 mothers of normally developing children of the same age. Research methods: Guilt Inventory (Jones &Kugler); Inventory of Parental Attitude (Varga &Stolin); Inventory of the Parent’s Psychological Type(Tkacheva).

**Results:**

The mothers of children with special needs show the more pronounced sense of guilt comparing with the mothers of the healthy children. They are less optimistic towards the future of the child, more sensible to the failures of the child, but demonstrate the higher degree of readiness to cooperation with the child. Those results can be applied when designing the intervention programs for the families of children with special needs.

**Conclusions:**

Those results can be applied when designing the intervention programs for the families of children with special needs.

**Disclosure:**

No significant relationships.

